# Natural flavonoids silymarin and quercetin improve the brain distribution of co-administered P-gp substrate drugs

**DOI:** 10.1186/s40064-016-3267-1

**Published:** 2016-09-20

**Authors:** D. Ravikumar Reddy, Amit Khurana, Swarna Bale, Ramu Ravirala, V. Samba Siva Reddy, M. Mohankumar, Chandraiah Godugu

**Affiliations:** 1 Laboratory of Nano-Biology, Department of Regulatory Toxicology, National Institute of Pharmaceutical Education and Research (NIPER), Balanagar, Hyderabad, Telangana State 500037 India; 2 Department of Drug Metabolism and Pharmacokinetics, Syngene International Ltd, Bangalore, Karnataka India

## Abstract

P-glycoprotein (P-gp), a well known efflux transporter in the blood brain barrier inhibits the uptake of substrate drugs into brain. The main aim of this study is to evaluate the effect of natural product based P-gp inhibitors on brain penetration of various CNS drugs which are P-gp substrates. In this study, we have evaluated the inhibitory effects of natural bioflavonoids (quercetin and silymarin) on P-gp by using digoxin and quinidine as model P-gp model substrate drugs. In vitro inhibitory effects were evaluated in Caco-2 cell lines using digoxin as a model drug and in vivo P-gp inhibiting effect was evaluated in mice model using quinidine as model drug. The accumulation and bidirectional transport of digoxin in Caco-2 cells was determined in presence and absence of quercetin and silymarin. Elacridar was used as standard P-gp inhibitor and used to compare the inhibitory effects of test compounds. The apical to basolateral transport of digoxin was increased where as basolateral to apical transport of digoxin was decreased in concentration dependent manner in the presence of elacridar, quercetin and silymarin. After intravenous administration of P-gp inhibitors, brain levels of quinidine were estimated using LC-MS method. Increased brain uptake was observed with quercetin (2.5-fold) and silymarin (3.5-fold). Though the brain penetration potential of P-gp substrates was lower than that observed in elacridar, both quercetin and silymarin improved plasma quinidine levels. Caco-2 permeability studies and brain uptake indicate that both quercetin and silymarin can inhibit P-gp mediated efflux of drug into brain. Our results suggest that both silymarin and quercetin could potentially increase the brain distribution of co-administered drugs that are P-gp substrates.

## Background

P-gp is an established factor in altering the pharmacokinetics of several drugs. Based on the studies of P-gp knockout mice and from the investigations of the effect of P-gp inhibitors on normal rodents and humans, it is known that P-gp is capable of decreasing the oral bioavailability and brain distribution of drugs that are substrates of the P-gp efflux pump (Gallo et al. 2003). Presence of P-gp expression at the apical surfaces of brain capillary endothelial cells and gut enterocytes has been determined to limit the penetration of P-gp substrates such as ivermectin and cyclosporin A in to the respective organs (Kwei et al. 1999). Later studies have revealed that P-gp is also present in many normal tissues including liver, kidney etc. (Cordon-Cardo et al. 1990). The generation of mice with disrupted P-gp knock out (KO) genes confirmed the significant protective pharmacological function of P-gp in the Blood Brain Barrier (BBB) (Schinkel et al. 1994, 1996). Entry of several drugs including vinblastine, cyclosporine A, digoxin, dexamethasone into the brain has been regulated by P-gp (Schinkel et al. 1995). In P-gp knockout mice, the penetration of vinblastine into the brain was 7–46 folds higher when compared with wild type control (Van Asperen et al. 1996), encompasing the role of P-gp in preventing the entry of its substrates into the brain. A fraction of epileptic patients do not respond to commonly prescribed antiepileptic drugs because of limited delivery to the brain. Studies suggest that the failure is because of over expression of ATP-driven efflux pumps at the BBB (Löscher and Potschka 2005). Escitalopram, a P-gp substrate, brain delivery was increased by P-gp inhibition using cyclosporin and verapamil thereby resulted in enhanced antidepressant activity with threefold increased brain concentration (O’Brien et al. 2013). Elacridar is a selective inhibitor for both P-gp and breast cancer resistance protein (BCRP) (Hyafil et al. 1993). There are number of drugs which have been reported as dual substrates for P-gp and BCRP (de Vries et al. 2007). For the drugs to act in the CNS, sufficient drug delivery is pre requisite. So, the evaluation of drug candidate susceptibility for P-gp efflux is a crucial step in the development of novel therapeutics particularly in targeting CNS. Silymarin, a bioflavonoid, is used for more than 2000 years to treat a range of liver and gallbladder disorders, including hepatitis, cirrhosis, and jaundice, and to protect the liver against poisoning from chemical and environmental toxins, including snake bites, insect stings, mushroom poisoning, and alcohol. The major active component is silibinin/silybin, which is hepatoprotective and possesses P-gp inhibiting property (Gazak et al. 2007). Silymarin potentiated the doxorubicin cytotoxicity in P-gp positive cells by inhibiting P-gp ATPase activity (Zhang and Morris 2003b). Similar results were observed in another study where silymarin in combination with biochanin A (an iso flavone from red clover extract) potentially increased the daunomycin cytotoxicity and decreased the daunomycin efflux in resistant breast cancer cell line, MCF-7 ADR (Chung et al. 2005). The same combination increased the accumulation of digoxin and vinblastine in intestinal Caco-2 cells in a concentration dependent manner (Zhang and Morris 2003a).

Quercetin, another flavonoid, has multiple biological actions such as antioxidant, antiulcer, antiallergic and anticancer. Currently, it is in clinical trials for the treatment of cancer and may be a promising drug of choice in future (Lakhanpal and Rai 2007). Quercetin decreased the resistance and increased the sensitivity of vinblastine and paclitaxel dose dependently in KBVI cells (human cervical carcinoma) which have P-gp expression (Limtrakul et al. 2005). Cell lines of human pancreatic carcinoma EPP85-181RDB (resistant to daunorubicin) and EPP85-181P (sensitive to daunorubicin) were treated with quercetin and found that quercetin altered the function of P-gp and decreased the expression of ABCB1 in EPP85-181RDB cell line. Through these findings quercetin was considered as potential modulator of P-gp (Borska et al. 2010). P-gp substrates digoxin and quinidine were selected based on the physicochemical properties. Digoxin is a lipophilic drug (logP = 2.37) with low permeability (Caco-2, P_app_ 1.1 × 10^−6^ cm/s) and quinidine is another lipophilic drug with high permeablity (Caco-2 P_app_ 20.4 × 10^−6^ cm/s) (Collett et al. 2004). The main aim of this study is to evaluate the effect of natural product based P-gp inhibitors on brain penetration of various CNS drugs which are P-gp substrates. Pharmacological inhibition of these efflux transporters prevents the inadequate distribution of drugs to the brain.

## Methods

### Materials

Caco-2 cells were procured from ATCC, USA. Transwell 24-well inserts were procured from Corning (USA), Dulbecco’s modified eagles medium (DMEM) was from Gibco, 96 well plate, hydrophilic solvinert plates were purchased from Millipore, USA. Digoxin, quinidine, quercetin, silymarin and dimethyl sulfoxide (DMSO) were purchased from Sigma-Aldrich (USA) and all other chemicals were of HPLC grade.

### Cell culture

Caco-2 cells were seeded in transwell poly carbonate inserts (6 well, 0.4 µm pore size, Corning co-star Co.) at 70,000 cells per insert on the day of seeding. Cells were cultured in DMEM supplemented with 10 % fetal bovine serum and 1 % non essential amino acids (Gibco). All the cells were incubated at 37 °C in a humidified atmosphere with 5 % CO_2_ and 95 % air. For uptake studies, Caco-2 cells were seeded on to 0.7 cm^2^ dishes at a density of approximately 70,000 cells per dish and used for experiment. On the day of 21 TEER (Tran’s epithelial electrical resistance) value was measured using Epithelial Volt ohmmeter (world precision instruments) and observed TEER value as more than 500 Ω cm^2^ that reflects confluent monolayer with tight junctions.

### Animals

Male C57 mice weighing 30–35 g and of 6–8 weeks old were taken and the animal experiments were conducted in the animal research facility of Syngene International limited, Bengaluru, India. Animals were kept under a 12 h light/dark cycle with free access to water and food (Kumar et al. 2014). Animal study protocols were approved by the Syngene International organisational animal ethics committee, Bengaluru, India.

### Formulation

Dose volumes (5 mL/kg) of drugs were administered by i.v. route via tail vein injection. Quinidine formulation was prepared using 10 % Dimethylacetamide (DMAC), 40 % Polyethyleneglycol (PEG-400), 20 % Water and 30 % of Hydroxypropyl β-cyclodextrin (HPβCD). The elacridar formulation contained 10 % DMAC, 40 % PEG-400, 30 % HPβCD and 20 % water, quercetin and silymarin formulations were prepared using 2 % *N*-methyl-2-pyrolidone, 10 % ethanol, 30 % Polyethylene glycol 200 (PEG 200) and 58 % saline.

### Transport study

Transport experiments using Caco-2 cell monolayers were performed, Caco-2 cell monolayer’s with TEER value higher than 500 cm^2^ were washed with transport buffer (HBSS) containing 10 mM HEPES buffer. Transport buffer containing quercetin and silymarin (50 and 100 µM) solution was incubated for 30 min in apical chamber for apical to basal (AP to BL) assay and basal chamber for basal to apical (BL to AP) assay in a single plate. All solutions were prepared in dimethylsulphoxide (DMSO). The final concentration of DMSO in the wells was less than 0.1 %. After incubation, wells were washed with transport buffer. Transport buffer containing digoxin 10 µM, elacridar 5 µM + digoxin 10 µM, Silymarin 50 µM + digoxin 10 µM, Silymarin 100 µM + digoxin 10 µM, Quercetin 50 µM + digoxin 10 µM, Quercetin 100 µM + digoxin 10 µM was added to apical chamber for apical to basolateral assay and basal chamber for basolateral to apical assay, rest added with 2 % bovine serum albumin (BSA) solution for non-specific interaction to the assay. Samples (200 µL) were taken from the receiver side at both chambers for analysis after incubating the cell monolayer’s at 37 °C for 30, 60 and 90 min and replaced with fresh transport buffer. The Caco-2 cell viability was studied in presence of silymarin and quercetin and found that the cell viability was not affected.

### Brain penetration study of elacridar and quinidine

Mice were divided into two groups (n = 4). One group dosed with elacridar (5 mg/kg) i.v. 30 min prior to the dosing of quinidine (5 mg/kg) and the other group dosed with quinidine alone. Blood and brain samples were collected at 0.5, 1, 3, 5 and 7 h post dose of probe substrate quinidine. Plasma and brain samples were collected and stored at −80 °C until analysis.

### Brain penetration study of silymarin and quercetin with quinidine

Mice were divided into three groups (n = 3–4). First group dosed with silymarin (20 mg/kg) i.v. 30 min prior to the dosing of quinidine (5 mg/kg). Second group dosed with quercetin (20 mg/kg) i.v. 30 min prior to the dosing of probe substrate quinidine and the other group dosed with probe substrate quinidine alone. Blood and brain samples were collected at 0.5, 1, 3, 5 and 7 h post dose of probe substrate. Blood was collected from mice under mild anaesthesia via tail vein and collected blood was centrifuged at 5000 rpm to separate plasma.

### Sample preparation

After sacrificing the mice at different time points, brain was collected homogenised at 5× concentration with phosphate buffered saline using Bullet blender. Fifty µL of brain homogenate or plasma and 200 µL of internal standard (IS) in vehicle (70 % acetonitrile and 30 % water) were mixed in a 96-well hydrophilic solvinert plate. The acetonitrile mixtures were vortexed and centrifuged at 10,000 rpm for 10 min. Supernatant was collected in a 96 well plate and analysed by liquid chromatography with mass spectroscopy (LC-MS, QTRAP ABSCIEX API 4000).

### LC-MS analysis

Standard curves were prepared by spiking a known concentration of quinidine into blank matrix and then processed according to the procedures described previously for each sample. Analysis was carried out using 4000 QTRAP LC/MS/MS system with triple Quadruple mass spectrometer (AB SCIEX) equipped with an electron spray ionisation (ESI). The mass spectrometer was operated in the ESI positive ion mode and detection of ions were performed in the multiple reaction monitoring (MRM) mode. The system was run in a gradient mode and flow rate was set at 0.67 mL/min for runtime of 2.5 min (Table 1). The standard curves were linear and assay accuracy was found to be between 85 and 115 %.

**Table 1:** LC-MS conditions followed to analyze quinidine and digoxin in plasma and cell culture samples

Compound	MRM transition	DP	CE	Mobile phase and column
Digoxin	798.5/651.4	85	21	Mobile phase A 0.1 % acetonitrile in milli Q water Mobile phase B 0.1 % Formic acid in Acetonitrile Column Kinetex 50 mm (C_18_)
Quinidine	325.2/184.2	130	70	

*MRM* multiple reaction monitoring, *DP* declustering potential, *CE* collision energy

### Pharmacokinetic analysis

Pharmacokinetic parameters (C_max_, t_1/2_, AUC, T_max_) were determined based on non compartmental approach using Phoenix winNonlin (Version 6.3).

### Statistical analysis

The data obtained in this study were expressed as the mean of replicate determinations (n = 3–4) plus or minus the standard error mean (SEM). Statistical comparisons were made using T-test and one way analysis of variance (ANOVA). The intergroup variations were measured by Bonferroni’s Mulptiple comparison test using the software Graph Pad Prism 5.0.

## Results

### Effect of elacridar on the transport of P-gp substrate digoxin across Caco-2 monolayers

**Fig. 1:**
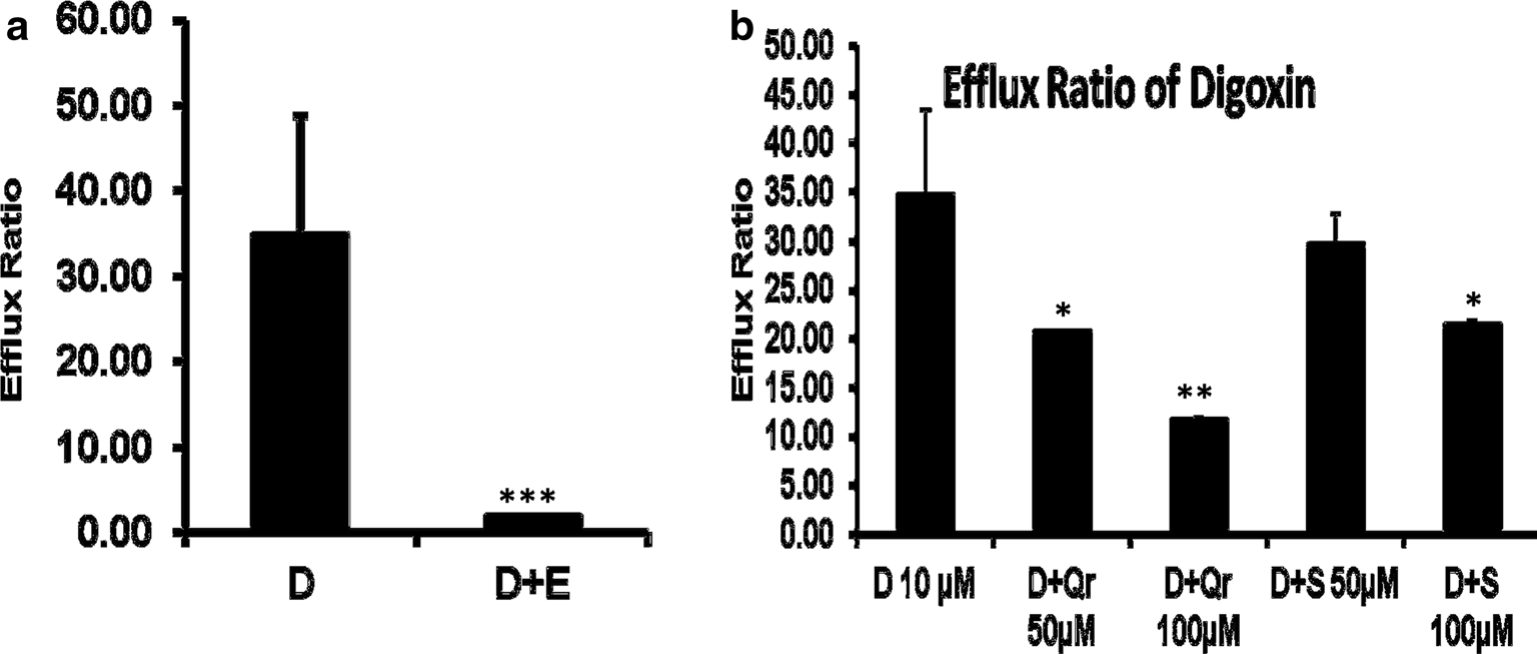
In vitro Caco-2 based P-gp inhibition study: **a** Efflux ratio of Digoxin and Digoxin with Elacridar, **b** Efflux ratio of Digoxin 10 µM alone, Digoxin in presence of Quercetin and Silymarin (50 and 100 µM). Each data point was represented as mean ± SEM (*n* = 3–4). *p < 0.05; **p < 0.01 and ***p < 0.001 vs Quinidine alone groups. (*D* digoxin, *E* elacridar, *Qr* quercetin, *S* silymarin)

Elacridar was standardised on the transport of digoxin across Caco-2 cell monolayer’s in both apical to basolateral and basolateral to apical directions. As shown in Table 2, the apparent permeability coefficient for basolateral to apical transport of digoxin (P_appB–A_: 1.8 × 10^−6^ cm/s) was higher than apical to basolateral (P_appA–B_: 5.18 × 10^−8^ cm/s) with a mean transport ratio (P_appB–A_/P_appA–B_) of 34.77, which shows the involvement of P-gp mediated efflux of digoxin in these cells. In presence of 5 µM elacridar (Table 2), the digoxin efflux of P_appA–B_ was significantly increased (from 5.18 × 10^−8^ to 1.52 × 10^−7^ cm/s) whereas the P_appB–A_ was significantly decreased (from: 1.8 × 10^−6^ to 2.78 × 10^−7^ cm/s) resulting into a mean transport ratio of 1.83 (Table 2). All these results suggest that elacridar inhibited P-gp mediated cellular efflux and thus increase the apical to basolateral transport of digoxin and decrease the basolateral to apical transport of digoxin, which indicates that elacridar as a complete P-gp inhibitor (Table 2; Fig. 1).

**Table 2:** Efflux ratio for digoxin in presence and absence of elacridar in the Caco-2 cell lines

Treatment	AP-BL transport	BL-AP transport	Efflux ratio (BL-AP/AP-BL)
Digoxin10 µM	5.18E−08	1.80E−06	34.77 ± 6.7
Digoxin + Elacridar 5 µM	1.52E−07	2.79E−07	1.83 ± 0.08

### Effect of silymarin and quercetin on the transport of P-gp substrate digoxin across Caco-2 monolayers

As shown in Table 3, the apparent permeability coefficient for basolateral to apical transport of digoxin (P_appB–A_: 1.8 × 10^−6^ cm/s) was higher than apical to basolateral (P_appA–B_: 5.18 × 10^−8^ cm/s) with a mean transport ratio (P_appB–A_/P_appA–B_) of 34.77, which shows the involvement of P-gp mediated efflux of digoxin in these cells. In presence of 50 µM quercetin, the P_appA–B_ was slightly increased (from 5.18 × 10^−8^ to 5.93 × 10^−8^ cm/s) whereas the P_appB–A_ was slightly decreased (from 1.8 × 10^−6^ to 1.23 × 10^−6^ cm/s) resulting a mean transport ratio of 20.79. In presence of 100 µM quercetin (Table 3), the P_appA–B_ was increased (from 5.18 × 10^−8^ to 1.04 × 10^−7^ cm/s) whereas the P_appB–A_ was decreased (from 1.8 × 10^−6^ to 1.21 × 10^−6^ cm/s) resulting a mean transport ratio of 11.70. In presence of 50 µM silymarin, the P_appA–B_ was increased (from 5.18 × 10^−8^ to 5.51 × 10^−8^ cm/s) whereas the P_appB–A_ was decreased (from 1.8 × 10^−6^ to 1.63 × 10^−6^ cm/s) resulting in a mean transport ratio of 29.69 In presence of 100 µM silymarin, the P_appA–B_ was increased (from 5.18 × 10^−8^ to 6.64 × 10^−8^ cm/s) whereas the P_appB–A_ was decreased (from 1.8 × 10^−6^ to 1.42 × 10^−6^ cm/s) resulting an mean transport ratio of 21.35 (Table 3). The Caco-2 permeability results indicate that both silymarin and quercetin can inhibit P-gp mediated cellular efflux and thus increase the AP to BL transport of digoxin and decrease the BL to AP transport of digoxin. Quercetin had stronger effects than silymarin when used at the same concentration, but neither of the flavonoids blocked P-gp completely at the tested concentrations of quercetin and silymarin at 50 and 100 µM respectively. Both silymarin and quercetin showed concentration dependent effect on digoxin transport as shown in the Table 3 and Fig. 1.

**Table 3:** Efflux ratio for digoxin in presence and absence of silymarin and quercetin in the Caco-2 cell lines

Treatment	AP-BL transport 10^−8^ cm/s	BL-AP transport 10^−6^ cm/s	Efflux ratio (BL-AP/AP-BL)
Digoxin (10 µM)	5.18	1.80	34.77 ± 14.02
Digoxin + Quercetin (50 µM)	5.92	1.23	20.79 ± 0.13
Digoxin + Quercetin (100 µM)	10.4	1.22	11.70 ± 0.21
Digoxin + Silymarin (50 µM)	5.51	1.64	29.69 ± 3.10
Digoxin + Silymarin (100 µM)	6.64	1.42	21.35 ± 0.50

### Effect of elacridar on plasma and brain pharmacokinetics and brain penetration of quinidine

Results of in vitro study motivated us to evaluate the efficacy in vivo. Mouse mean plasma concentration versus time of i.v. dosed quinidine (5 mg/kg), in the presence and absence of elacridar (5 mg/kg i.v. 0.5 h pre-treatment) were recorded. Pre-treatment of elacridar showed no significant difference in the plasma profile of quinidine, but a trend of increased t_1/2_ was observed whereas pre-treatment with elacridar resulted in significant difference in the brain profile of quinidine concentration with an increased C_max_ and brain area under the curve (AUC) _0–7h_ (Table 4).

**Table 4:** Mean plasma and brain pharmacokinetics parameters of quinidine in the presence and absence of elacridar after i.v. administration in mice

Parameters	Plasma pharmacokinetics		Brain pharmacokinetics	
	Quinidine (5 mg/kg)	Quinidine with elacridar (5 mg/kg)	Quinidine (5 mg/kg)	Quinidine with elacridar (5 mg/kg)
Dose (mg/kg)	5	5	5	5
Half life (h)	1.43	2.15	1.50	0.88
T_max_ (h)	0.66	0.50	0.80	0.83
C_max_ (ng/mL)	209.16	185.87	115.40	2225.80
AUClast (h ng/mL)	496.40	459.80	211.40	4998.65
AUCINF_obs (h ng/mL)	513.40	523.83	251.20	5039.93

### Effect of silymarin and quercetin on plasma and brain pharmacokinetics and brain penetration of quinidine

**Fig. 2:**
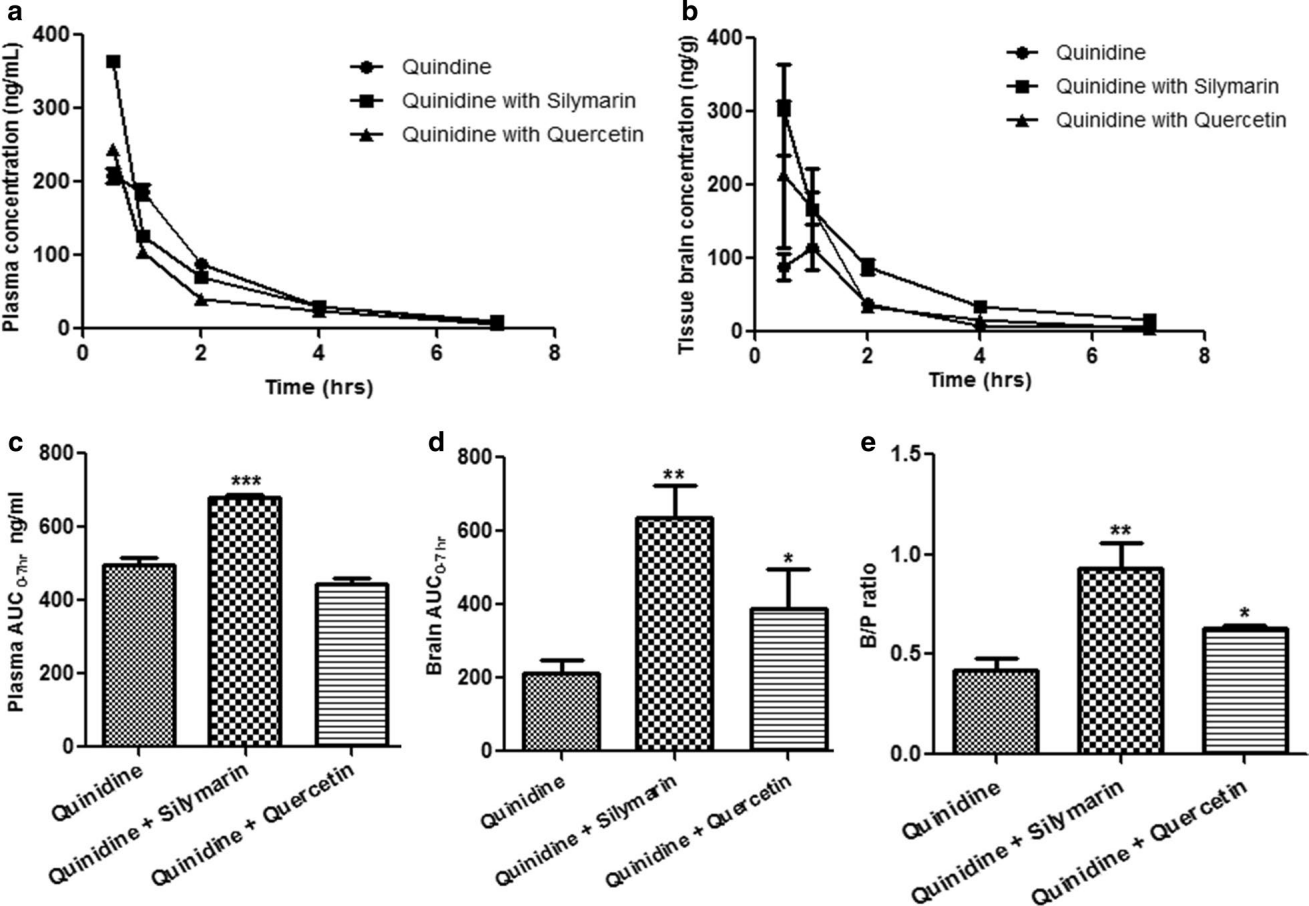
In vivo P-gp inhibition study: **a** Mean plasma concentration, **b** Mean brain concentration, **c** Plasma AUC. **d** Brain AUC and **e** Brain to plasma ratio of Quinidine when combined with Quercetin and Silymarin. Each data point was represented as mean ± SEM (n = 3–4). *p < 0.05; **p < .0.01 and ***p < 0.001 versus Quinidine alone groups

**Fig. 3:**
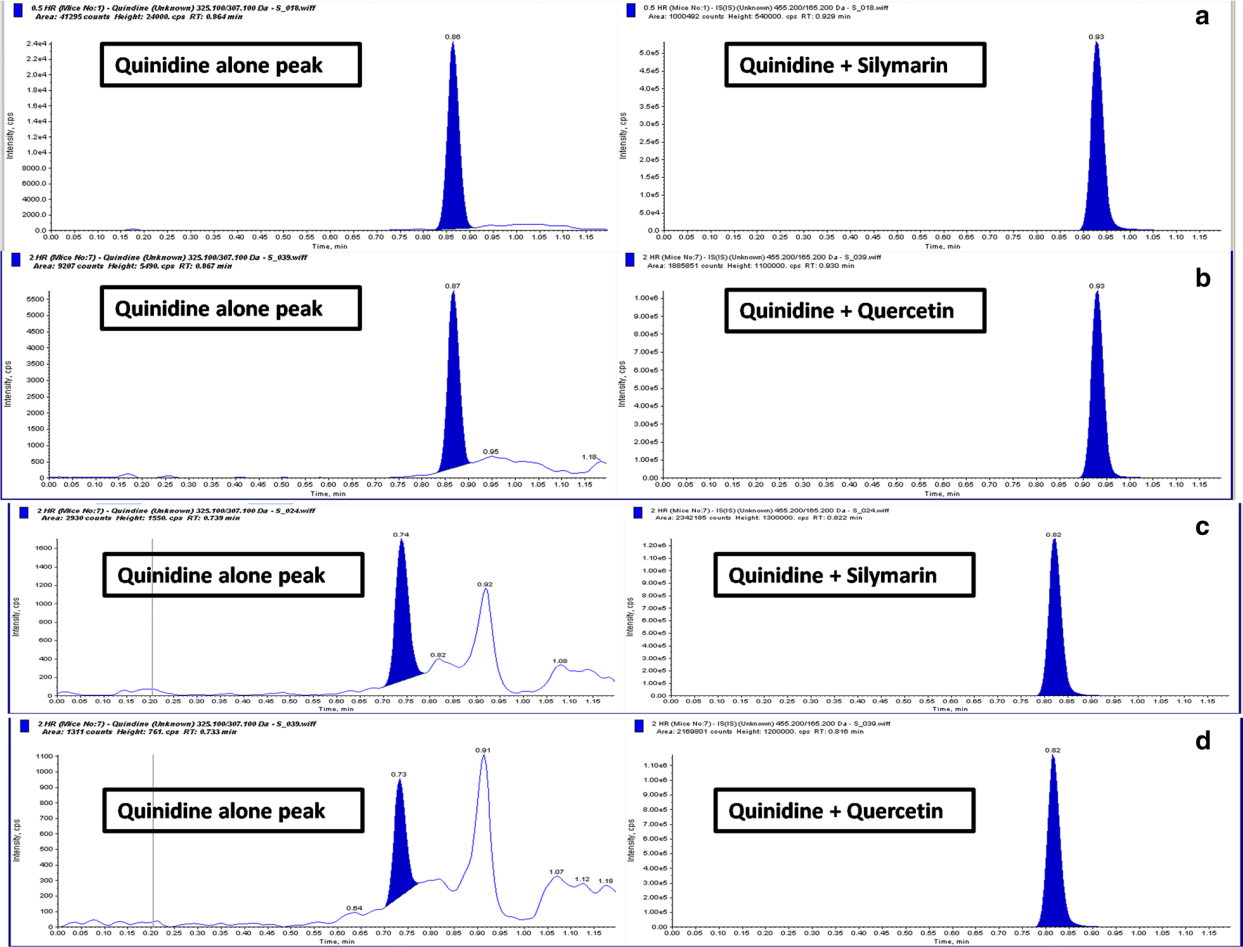
Representative LC-MS chromatograms of Quinidine, **a** plasma concentration of Quinidine + Silymarin; **b** plasma concentration of Quinidine + Quercetin; **c** brain concentrations of Quinidine + Silymarin, and **d** brain concentrations of Quinidine + Quercetin after 2 h of administration. The *left lane* peaks are quinidine alone and *right lane* in presence of Silymarin/Quercetin. The shift in retention time in respective chromatograms indicates significant increase in the area under the curve in comparison to the quinidine alone peaks indicating effective P-gp inhibition in presence of drug treatment

Pre-treatment of silymarin showed significant improvement in plasma pharmacokinetic profile as well as brain penetration of quinidine with increased t_1/2_, C_max_ and AUC. Pre-treatment of silymarin resulted in a maximum 3.5-folds increase in Kp, brain of quinidine. Pre-treatment of silymarin increased (AUC) _0–7h_ B/P by twofolds for quinidine (Fig. 2; Table 5). However, pre-treatment of quercetin showed no significant difference in plasma profile, but a trend of increased t_1/2_ and C_max_ was observed indicating an increased brain profile of quinidine with a trend of increased t_1/2_, C_max_ and AUC. In contrast, pre-treatment with quercetin resulted in 2.5-folds increase in Kp and brain (AUC) _0–7h_ B/P by twofold of quinidine. The respective representation of chromatograms depicting concentration of quinidine, quinidine + silymarin and quinidine + quercetin in both plasma and brain are mentioned in Fig. 3.

**Table 5:** Mean plasma and brain pharmacokinetic parameters of quinidine in the presence and absence of silymarin and quercetin (10 mg/kg) after i.v. administration in mice

Parameters	Plasma pharmacokinetics			Brain pharmacokinetics		
	Quinidine	With silymarin	With quercetin	Quinidine	With silymarin	With quercetin
Dose (mg/kg)	5	10	10	5	10	10
Half life (h)	1.43	1.73	1.86	1.50	1.95	1.91
T_max_ (h)	0.66	0.50	0.50	0.80	0.50	0.83
C_max_ (ng/mL)	209.16	364.48	244.80	115.40	301.60	235.73
AUClast (h ng/mL)	496.40	680.43	445.29	211.40	635.15	390.09
AUCINF_obs (h ng/mL)	513.40	704.07	463.55	251.20	682.40	405.64

## Discussion

Delivery to brain has ever been a major hurdle by virtue of blood brain barrier (BBB) that shields the brain from other body organs. Treatment of CNS disorders like epilepsy, Alzheimer and brain tumors poses a great challenge due to sub effective concentration of drug reaching the target site. To achieve this goal, the researchers are working on novel strategies to improve the BBB distribution of drugs in glioblastoma patients. The present study aims at improvement of brain delivery of quinidine, an FDA approved drug used for cerebral malaria and a well known P-gp substrate, by using natural flavonoids quercetin and silymarin, which are well known P-gp inhibitors. Several lines of evidences have reported the role of both quercetin and silymarin as intestinal P-gp inhibitors. Significant improvement of relative bioavailability was observed in study performed to enhance the oral bioavailability of paclitaxel and its formulation (Taxol) using silymarin and quercetin as P-gp inhibitor (Park et al. 2012; Choi et al. 2004). In consistent with the findings, in present study we used silymarin and quercetin to inhibit the P-gp at the BBB. The presence of P-gp efflux transporter at the BBB may restrict the entry of several P-gp substrates into the brain. The in vivo brain-to-plasma concentration ratio of vincristine in ddY mice was decreased by co-administration of 0.1 mg/kg quercetin, but increased by 1.0 mg/kg quercetin (Mitsunaga et al. 2000). In current study, we used different drugs/agents either as P-gp substrates (quinidine and digoxin) and P-gp inhibitors (elacridar, silymarin and quercetin) to evaluate the role of P-gp in brain distribution of drugs.

The model was established with standard P-gp inhibitor elacridar and was used to analyze the brain penetration of quinidine. In vitro Caco-2 cell line based permeability studies were performed by using digoxin as model P-gp substrate. The model P-gp subtrates, quinidine (high permeabililty) and digoxin (low permeability) were selected, which are lipophilic drugs and having Caco-2 P_app_ 20.4 × 10^−6^ and 1.1 × 10^−6^ cm/s, respectively (Schinkel et al. 1995). The effect of in vivo P-gp inhibition of elacridar, silymarin and quercetin was calculated in terms of the increase in K_pbrain_ and brain to plasma AUC ratio in mice model. The in vitro P-gp inhibition was studied as efflux ratio of probe substrates in presence of selected inhibitors in Caco-2 cell line. Further, several studies had shown that P-gp efflux transporter contributes to the efflux of digoxin and quinidine across the BBB (Mayer et al. 1997).

We estimated P-gp inhibition of quercetin and silymarin using Caco-2 permeability assay and compared those inhibitory effects with standard strong P-gp inhibitor elacridar. Both quercetin and silymarin produced concentration dependent P-gp inhibitory effects on Caco-2 cell lines by increasing the apical to basolateral transport of digoxin. We observed a significant change in the transport of digoxin with both the selected test inhibitors at 50 and 100 µM concentrations for AP-to-BL as well as BL-to-AP transport. By using quercetin as a P-gp inhibitor similar type of results were published by Zhang et al. (Zhang and Morris 2003a). In our study, quercetin appears to be more potent than silymarin in terms of digoxin efflux ratio on Caco-2 cell lines. K_pbrain_ of P-gp substrate quinidine was measured at different time point’s *i.e.* 0.5, 1, 2, 4 and 7 h in mice in the presence of elacridar, quercetin and silymarin. At 1 h time point, the fold increase in K_pbrain_ of P-gp substrate quinidine was found to be 22.5 in presence of elacridar, 3.5 in the presence of silymarin and 2.5 in the presence of quercetin. These results suggest that K_pbrain_ values varied with time of measurement and distribution kinetics of the compound. A single time point measurement could mislead the evaluation of brain penetration of P-gp substrate. Therefore, in addition to K_pbrain_ we also determined the brain to plasma AUC ratio (B/P).

Quinidine showed a 3.5 and 2.5 folds increase in B/P ratio in combination with silymarin and quercetin, respectively in mice. Similar results were published by Xiao et al. (2012) and Batrakova et al. (2001). In presence of elacridar in mice, the B/P ratio of quinidine was increased by 22.5 fold over the control animals. Similarly, Kallem et al. (2012) reported a 38 fold increase in the B/P of quinidine in mice. The greater improvement of quinidine B/P with elacridar was compared with that of silymarin and quercetin. It was known that compounds with B/P greater than two in mdr1a/1b KO over the wild type mice (WT) are P-gp substrates (Liu et al. 2009). In the present study, animals treated with elacridar, silymarin and quercetin also resulted in B/P ratio greater than two which confirms that selected inhibitors have significant impact on the brain distribution of P-substrate drugs like quinidine. Moreover, findings of Youdim *et al.*, prove that quercetin is able to effectively traverse the BBB based on the rate of uptake in in vitro (ECV304/C6 coculture) and in situ (rat, cerebral hemispheres) models. Quercetin showed measurable in vitro and in situ BBB permeability. Furthermore, quercetin showed measurable quantities inside MDCK-MDR1 and immortalized rat brain endothelial cells (RBE4) proving the fact that it is able to bypass the over expressed efflux transporters, which is in line with our in vivo findings (Ishisaka et al. 2011). In another study Mitsunaga et al. (2000) showed the increase in uptake of [^3^H]vincristine across BBB upon treatment with 50 μM quercetin in vitro in cultured mouse brain capillary endothelial cells (MBEC4) and by 1.0 mg/kg quercetin in vivo (ddY mice) which further conceptually proves our hypothesis. We also examined the effect of silymarin and quercetin on the pharmacokinetics of quinidine in mice and found an increase in half life and AUC compared with quinidine control.

Our study described that co-administration of silymarin and quercetin can improve drug delivery of P-gp substrate drugs in several brain disorders including glioblastoma and epilepsy. The P-gp inhibitory effects of quercetin and silymarin can be beneficial to deliver the drugs into brain where P-gp mediated efflux is the major barrier. Both, quercetin and silymarin have numerous pharmacological activities and can synergise with many of such drugs that face the problem of poor brain penetration.

## Conclusion

We conclude that concurrent use of quercetin and silymarin is safe in combination with the drugs which are P-gp substrates, to increase brain distribution. These compounds possess multiple pharmacological actions like anti-oxidant, anti-cancer, and anti-fibrotic activities, thus can be used synergistically for efficacious therapy with other conventional treatment regimens. Besides, the anti-oxidant nature of these drugs will nullify the oxidative stress produced by the P-gp substrate drugs like doxorubicin etc which further adds to the significance of the use of anti-oxidants of plant origin.
